# Radiotherapy in the management of PDAC, from past to present

**DOI:** 10.1016/j.tranon.2025.102632

**Published:** 2025-12-08

**Authors:** Julie Dardare, Nicolas Martz, Andréa Witz, Margaux Betz, Cassandra Michel, Pauline Gilson, Jean-Louis Merlin, Aurélien Lambert, Alexandre Harle

**Affiliations:** a Service de Médecine de Précision et Recherche Translationnelle, Institut de Cancérologie de Lorraine, Vandœuvre-lès-Nancy, France; b Academic Department of Radiation Therapy & Brachytherapy, Institut de Cancérologie de Lorraine, Vandoeuvre Les Nancy, France; c Université de Lorraine, Centre National de la Recherche Scientifique (CNRS), Unité Mixte de Recherche (UMR) 7039 Centre de Recherche en Automatique de Nancy (CRAN), Nancy, France

**Keywords:** Pancreatic ductal adenocarcinoma, Radiotherapy, Chemoradiotherapy, Clinical trials

## Abstract

Pancreatic ductal adenocarcinoma (PDAC) remains one of the most lethal forms of cancer, with a dismal survival rate. Therapeutic options are restricted in surgery for patients with resectable disease and in chemotherapy for those with unresectable disease. The potential benefits of radiotherapy (RT) or chemoradiotherapy (CRT) have been extensively investigated in both neoadjuvant and adjuvant settings in the management of patients with PDAC. Nevertheless, a substantial number of clinical trials have yielded conflicting findings, thereby rendering the impact of RT on patient survival and margin-negative (R0) resection inconclusive. A comprehensive examination of the historical evolution of RT in PDAC, encompassing the identification of both constraints and opportunities for advancement, is essential to establish RT as a promising therapeutic avenue for patients with PDAC. The aim of this review is to provide a synthesis of past clinical trials and future studies to elucidate the evolving role of RT in the management of PDAC as adjuvant and neoadjuvant CRT across different stages of the disease.

## Highlights

• The role of radiotherapy in the management of patients with pancreatic cancer remains a topic of ongoing debate.
• The available evidence suggests that adjuvant chemoradiotherapy is less effective than chemotherapy.
• Further investigation is required to ascertain the potential of neoadjuvant chemoradiotherapy in terms of the local disease control.
• Futures clinical trials should investigate modern radiotherapy using innovative techniques and more appropriate chemotherapy regimens in order to improve patient outcomes.

## Graphical abstract

**Figure:**
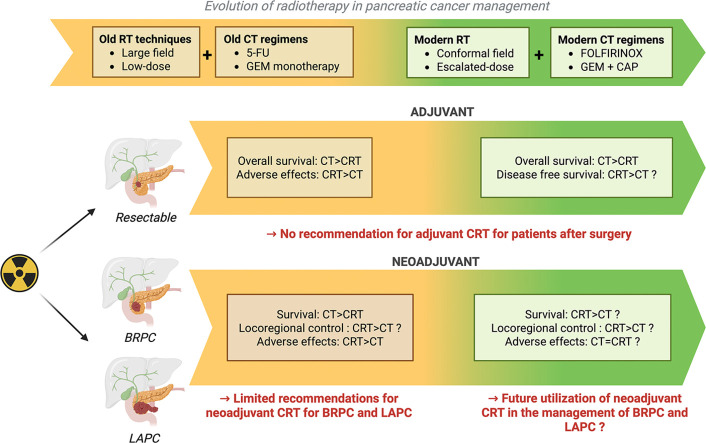

## Introduction

Pancreatic ductal adenocarcinoma (PDAC) is one of the deadliest cancers, with a 5-year relative survival rate of only 13 % for all stages combined [1]. Despite significant efforts deployed to understand the biology of pancreatic cancer and develop new treatments, PDAC remains a public health challenge. The management of PDAC patients depends on the tumor resectability [2]. Only 20 % of patients are diagnosed with a resectable localized tumor and can benefit from potentially curative treatment, which consists of surgical resection followed by adjuvant therapy [3]. For patients with unresectable disease at diagnosis, including borderline resectable pancreatic cancer (BRPC) and locally advanced pancreatic cancer (LAPC), therapeutic options consist of neoadjuvant treatment, primarily chemotherapy (CT), which may be administered in conjunction with radiotherapy (RT). The purpose of neoadjuvant treatment is to optimize local control and enhance the probability of achieving secondary R0 surgical resection. Among CT agents employed in adjuvant and neoadjuvant contexts, gemcitabine [4] and 5-fluorouracil (5-FU) [5] were the first monotherapies to be approved. The standard of care has since evolved to combination CT, including modified folinic acid, 5-FU, irinotecan, and oxaliplatin (mFOLFIRINOX) [[6], [7], [8]], gemcitabine plus nab-paclitaxel [9] or gemcitabine plus capecitabine [10].

The use of RT or chemoradiotherapy (CRT) in the management of PDAC patients has been widely debated due to conflicting results from several clinical trials. Although RT has been studied in both neoadjuvant and adjuvant settings, its effect on survival and margin-negative (R0) resection remains equivocal [5,[11], [12], [13], [14]]. Current guidelines on radiation therapy from the American Society of Clinical Oncology (ASCO) and the European Society for Medical Oncology (ESMO) are not entirely consistent. The latest ESMO clinical guidelines for PDAC do not recommend CRT as an adjuvant treatment for patients following surgery, nor in LAPC patients. However CRT can be considered after induction CT in patients with BRPC [15]. On the other hand, ASCO guidelines suggest the use of adjuvant CRT for resected patients who are preoperative therapy-naive with microscopically positive margins (R1) and/or have node-positive disease after completion of systemic adjuvant CT [16]. Furthermore, ASCO provides three different recommendations for the use of CRT in LAPC patients: [1] in case of local disease progression after induction CT without systemic spread, [2] in case of response to 6 months of CT or stable disease, or with unacceptable CT-related toxicities, and [3] as an alternative to continuing CT alone in case of response or stable disease after 6 months of induction CT [17]. Given these controversies, revisiting the historical evolution of RT and its influence on current standards is essential to contextualize emerging strategies.

The objective of this review is to provide a comprehensive examination of the historical evolution of RT in PDAC, including a detailed account of previous clinical trials investigating the use of adjuvant RT for resected patients, as well as the use of neoadjuvant RT in the treatment of both BRPC and LAPC patients. A deeper comprehension of the obstacles and levers of past studies may contribute to the enhancement of future clinical trials, thereby establishing RT as a promising therapeutic avenue for patients with PDAC.

## Adjuvant radiotherapy

### Early trials and historical controversies

The use of RT in the management of PDAC has been a topic of discussion for decades. The Gastrointestinal Tumor Study Group (GITSG) conducted the first and only prospective randomized trial demonstrating an advantage for adjuvant CRT. The study compared adjuvant treatment with 5-FU-based CRT followed by maintenance CT (40 Gy split into two courses with concurrent bolus 5-FU, followed by maintenance 5-FU for 2 years or until disease progression) to surgery alone in 43 patients with pancreatic cancer. The median overall survival (OS) increased from 11 months in the surgery group to 20 months in the adjuvant group. Additionally, the adjuvant group exhibited a higher 2-year survival rate of 43 % (confidence interval 95 % (95 % CI): 0.25 – 0.63) compared to the surgery group’s rate of 18 % (95 % CI: 0.08 – 0.36; *p* < 0.03) [18,19] (Table 1). These results were confirmed in a validation cohort of 30 additional patients who received the same treatment and exhibited a similar median OS of 18 months [19]. Although previous studies have suggested the benefits of using 5-FU-based CRT as adjuvant therapy after curative resection, subsequent studies have failed to confirm these observations. A phase III clinical trial initiated by the European Organization for Research and Treatment of Cancer (EORTC) found no advantage in terms of progression-free survival (PFS) or OS duration for adjuvant 5-FU-based CRT (40 Gy split into two courses and concurrent infusional 5-FU, without maintenance CT) compared to observation after surgery [20]. In a same manner, the multicenter randomized ESPAC-1 trial, conducted by the European Study Group for Pancreatic Cancer (ESPAC), included 541 eligible patients who were randomized in a two-by-two factorial design: CT (5-FU) *versus* no CT or CRT *versus* no CRT (20 Gy in 10 fractions with concurrent bolus 5-FU). The results revealed a deleterious effect on the survival for 5-FU-based CRT, with a 5-year survival rate of 10 % compared to 20 % for patients who did not receive CRT (*p* *=* *0.05*). The authors concluded that the standard of care for patients with resectable PDAC should consist of surgery followed by adjuvant systemic CT, based on a significantly longer 5-year survival rate of 21 % for CT compared to 8 % for patients who did not received CT (*p* *=* *0.009*) [5]. Adjuvant 5-FU-based CRT was investigated with either 5-FU or gemcitabine in pre- and post-CRT in the NRG/Radiation Therapy Oncology Group (RTOG) 9704 trial. The initial 3-year analysis revealed a survival benefit for resected PDAC patients with the addition of gemcitabine to adjuvant 5-FU-based CRT [21]. However, this observation failed to be confirmed in the 5-year results [22]. The addition of other CT agents, such as cisplatin and interferon alfa-2b (IFN α−2b), was associated with 5-FU-based CRT. A phase III trial showed that CRT regimen did not improve median survival compared to 5-FU alone (26.5 months *versus* 28.5 months; *p* *=* *0.99*), with a high rate of adverse effects [23]. While early prospective trials provided conflicting results, subsequent retrospective studies attempted to clarify RT’s impact by leveraging larger patient cohorts and real-world data.

**Table 1:** Clinical trials evaluating CRT as adjuvant setting in the management of resected PDAC.

Trial	Setting	Treatments	Patient outcomes	Main conclusion
GITSG (Randomized) [18]	Adjuvant 5-FU-based CRT *vs* surgery alone for resected pancreatic cancer.	CRT (40 Gy in 20 fractions plus 5-FU) (*n* = 21) *vs* surgery alone (*n* = 22).	Median OS: CRT 20 months *vs* surgery alone 11 months (*p=0.03*).	Benefit of adjuvant 5-FU based CRT.
EORTC trial (Phase III) [20]	Adjuvant 5-FU-based CRT *vs* surgery alone for resected pancreatic cancer.	CRT (40 Gy in 20 fractions plus 5-FU) (*n* = 110) *vs* surgery alone (*n* = 108).	Median duration of survival: CRT 24.5 months *vs* surgery 19.0 months (*log-rank p = 0.208*). 2-year survival rate: CRT 34 % *vs* surgery 26 % (*log-rank p = 0.099*).	No benefit of CRT.
ESPAC-1 (Randomized) [5]	Adjuvant 5-FU-based CRT *vs* adjuvant CT for resected pancreatic cancer.	CRT (20 Gy in 10 fractions plus 5-FU) (*n* = 145) *vs* no CRT (*n* = 144). CT (leucovorin plus 5-FU for 6 cycles) (*n* = 147) *vs* no CT (*n* = 142).	Median OS: CRT 15.9 months (95 % IC: 13.7 to 19.9) *vs* no CRT: 17.9 months (95 % IC: 14.8 to 23.6) (HR: 1.28; 95 % CI: 0.99 - 1.66; *p=0.05*). Median OS CT 20.1 months (95 % CI: 16.5 - 22.7) *vs* no CT:15.5 months (95 % CI: 13.0 - 17.7) (HR: 0.71; 95 % CI: 0.55 - 0.92; *p=0.009*).	Benefits with adjuvant CT. CRT failed to benefit patients and reduces survivals.
RTOG 97–04 (Phase III) [21,22]	Adjuvant 5-FU-based CRT with either 5-FU or gemcitabine in pre- and post-CRT for resected PDAC.	CRT (50.4 Gy in 28 fractions) with pre and post 5-FU (*n* = 230) or GEM (*n* = 221).	Median survival of 20.5 months and a 3-year OS of 31 % in GEM group *vs* 16.9 months and 22 % in 5-FU group (HR: 0.82; 95 % CI: 0.65–1.03; *p= 0.09).* Median survival and 5-year OS of 20.5 months and 22 % in GEM group *vs* 17.1 months and 18 % in 5-FU group (HR: 0.84; 95 % CI: 0.67 – 1.05; *p=0.12*).	Improvement in 3-year OS with the addition of GEM to adjuvant CRT but no longer seen at 5 years.
CapRI trial (Phase III) [23]	Adjuvant 5-FU and cisplatin-based CRT plus Interferon Alfa-2b *vs* CT for resected pancreatic cancer.	CRT (50.4 Gy in 28 fractions plus 5-FU, cisplatin and IFN α−2b) (*n* = 64) *vs* CT (5-FU plus folinic acid) (*n* = 68).	Median OS: CRT 26.5 months (95 % CI, 21.6 - 39.5 months) *vs* CT 28.5 months (95 % CI, 20.4 - 38.6 months) (HR:1.04; 95 % CI: 0.66 - 1.53; *P* = 0 0.99).	No improvement of survival of CRT and more adverse effects.
NRG/RTOG 0848 [28,29]	Adjuvant CRT in resected PDAC after adjuvant GEM with or whithout Erlo.	First randomization: GEM(*n* = 163) *vs* GEM + Erlo (*n* = 159) Second randomization: CRT (50.4 Gy in 28 fractions) (*n* = 180) *vs* CT (*n* = 174)	First randomization: Median OS: GEM 29.9 (95 % CI: 21.7 – 33.4) months *vs* GEM + Erlo 28.1 months (95 % CI: 20.7 – 30.9) (log-rank *p* = 0.62). Second randomization: Median OS: CT 31 months (90 % CI: 25–38) *vs* CRT 27 months (90 % CI: 25 – 32) (log-rank *p* = 0.38) Median DFS: CT 12 months (90 % CI: 9 – 18) *vs* CRT 16 months (90 % CI: 12 – 20) (log rank *p* = 0.045)	No benefits of Erlo combined with GEM. Improvement of DFS with CRT *vs* CT.

5-FU, 5-fluorouracil; CRT, chemoradiotherapy; CT, chemotherapy; GEM, gemcitabine; HR, hazard ratio; OS, overall survival; erlo, erlotinib.

### Retrospective analyses and quality concerns

Retrospective studies that encompassing a large sample of patients have demonstrated that adjuvant CRT in resected PDAC patients provides benefits in terms of survival compared to surgery alone [24,25]. A recent retrospective study of patients with PDAC who underwent R1 resection reported survival outcomes that were marginally higher than those previously reported for patients receiving adjuvant CT followed by CRT (50.4–54 Gy) [26]. Nevertheless, retrospective studies may be subject to bias due to nonrandom allocation of patients to treatment, which can weaken the strength of their evidence. It is important to note that these previous randomized studies have also notable limitations. For example, the GITSG study had a small sample size of only 43 participants and used an outdated split course RT. The EORTC study has been criticized for its statistical methodology with using a two-sided log-rank test instead of a one-sided analysis. Indeed, a reanalysis of the results shown significantly higher survival in the CRT arm (*p* *=* *0.049*)[27]. In addition, this trial did not require RT quality assurance. The ESPAC-1 trial had several limitations, including the possibility of administering background adjuvant therapy prior to randomization, the lack of central review, and the lack of standardization of RT dose and fields. However, as it has been demonstrated in the NRG/RTOG 9704 study, there is a strong correlation between radiation quality with the survival of patients. The endless discussion about adjuvant RT led clinicians to move away from using adjuvant CRT for PDAC patients especially in Europe. These retrospective insights, despite their limitations, paved the way for modern prospective trials using improved RT techniques.

### Modern trials and ongoing evidence

The NRG/RTOG 0848 phase III randomized trial (NCT01013649) may help to establish a statement about adjuvant use of CRT. The trial initially included a first randomization to adjuvant gemcitabine with or without erlotinib, but this arm has been removed due to the absence of improvement in OS (29.9 *versus* 28.1 months; *p* *=* *0.62*) [28]. The second primary objective of this study is to determine whether fluoropyrimidine-based CRT (5-FU/capecitabine with 50.4 Gy in 28 fractions) can improve the survival of patients who have not experienced disease progression after five months of adjuvant gemcitabine. The preliminary results of this randomization indicated that CT followed by CRT did not improve OS, but did improve disease-free survival (DFS)(*p* *=* *0.045)*. Furthermore, both OS and DFS were improved with CT and CRT in node negative patients compared to CT alone (29 % *versus* 48 %; *p* *=* *0.0063,* and 19 % *versus* 47 %; *p* *=* *0.014*, respectively) [29]. The authors did not observe an increase in grade 4 or 5 adverse events with CT followed by CRT compared to CT alone. The publication of the final analysis is eagerly anticipated.

In recent years, novel methods of RT delivery have emerged, with the objective of enabling the delivery of high-dose radiation with minimal margins. Among these techniques, stereotactic body radiotherapy (SBRT) offers several advantages over conventional RT techniques, including a shorter treatment duration (1 week *versus* 4–6 weeks), a higher biological effective dose (BED), a more focused treatment field, and the capacity to spare organs at risk (OAR) more effectively. A paucity of clinical trials has explored the potential of SBRT in resected PDAC patients, yielding conflicting results. An initial retrospective study has demonstrated the safety and feasibility of adjuvant SBRT (20–24 Gy in one fraction) followed by systemic CT for resected PDAC patients with close (1–2.5 mm) or positive margins [30]. A subsequent prospective observational study corroborated these observations with adjuvant SBRT (36 Gy in 3 fractions) in 49 patients with positive or close margins (<2.5 mm). Despite the absence of substantial survival benefits, SBRT demonstrated a significant improvement in local and regional control accompanied by low rates of grade 3 toxicity [31]. In a prospective randomized single-center trial, the efficacy of adjuvant SBRT (25 Gy in 5 fractions) combined with gemcitabine has been compared with gemcitabine alone in margin-negative patients. The study indicated that the addition of SBRT in the adjuvant setting did not result in enhanced survival outcomes or improved local disease control [32].

Despite the paucity of evidence, the potential benefits of SBRT in achieving local control in patients with positive resection margins merit further investigation. Moreover, a notable benefit of this RT technique is its capacity for safe interdigitation with CT cycles, enabled by the delivery of adjuvant SBRT within a week. Further prospective trials are required to determine the value of SBRT in the adjuvant setting. In order to accomplish this objective, the ongoing phase II trial SPARTA (NCT05043857) is assessing the efficacy of adjuvant SBRT following surgery for PDAC [33]. The objective of this study is to deliver a dose-intensive treatment in a reduced time frame, thereby facilitating better integration of CT and surgery. Patients will receive two clinical target volumes (CTV) (CTV1 = 40 Gy in 5 fractions of 8 Gy, and CTV2 = 30 Gy in 5 fractions of 6 Gy). The results of this study are pending. The findings of contemporary clinical trials will be instrumental in determining the potential benefits of CRT in the adjuvant setting for patients with PDAC.

In summary, the role of adjuvant RT remains a matter of debate, while adjuvant CT continues to represent the standard of care (NCCN Guidelines 2025). However, according to the meta-analysis by Stocken et al., adjuvant CRT based on 5-FU may be considered after 3 to 6 months of adjuvant CT in cases of incomplete (R1) resection, following multidisciplinary tumor board approval [34]. If treatment is pursued, delivery of concurrent chemoradiation after 4–6 months of systemic CT is recommended [35].

## Neoadjuvant radiotherapy

Although surgical resection is the most effective therapeutic approach for PDAC, it cannot be used as the initial treatment for unresectable disease. Therefore, neoadjuvant treatments have emerged as the primary option for maximizing local control, increasing the likelihood of achieving secondary R0 surgical resection, prolonging OS, and avoiding unnecessary surgery in patients with evolving metastases [16]. Several prospective trials have investigated neoadjuvant therapies using CT or CRT for patients with BRPC and LAPC.

### Borderline resectable pancreatic cancer

#### Early trials

A first phase II/III randomized clinical trial (NCT01458717) has demonstrated the benefits of neoadjuvant CRT in BRPC patients. The trial enrolled a total of 58 patients and compared neoadjuvant gemcitabine-based CRT (45 Gy in 25 fractions and 9 Gy in 5 fractions) to immediate surgery followed by adjuvant CRT (same regimen as the neoadjuvant setting). In the intention-to-treat analysis, the neoadjuvant CRT arm showed a significantly higher 2-year OS of 21 months compared to 12 months for the upfront surgery arm (95 %IC: 0.66 – 3.36; *p* *=* *0.028*). The R0 resection rate was also significantly higher in the neoadjuvant CRT group than in the upfront surgery group (*p* *=* *0.004*). Furthermore, the neoadjuvant CRT arm had significantly smaller tumor size and fewer positive lymph nodes [36] (Table 2). The Dutch Pancreatic Cancer Group (DPCG) conducted the randomized phase III PREOPANC trial to compare upfront surgery with neoadjuvant gemcitabine-based CRT in 246 patients with resectable PDAC or BRPC. Preoperative CRT consisted of 3 cycles of gemcitabine combined with 36 Gy in 15 fractions RT, followed by surgery and 4 cycles of adjuvant gemcitabine. In the second arm, immediate surgery was followed by 6 cycles of adjuvant gemcitabine. Although the initial results failed to demonstrate a significant benefit in the primary endpoint of OS, the neoadjuvant CRT group had superior secondary endpoints such as DFS, locoregional failure-free interval, R0 resection rate, and pathologic lymph nodes. Specifically, the R0 resection rate was 72 % for the preoperative CRT group compared to 40 % for the surgery group (*p* *<* *0.001*) [37]. The long-term results have finally shown a benefit with neoadjuvant treatment compared to upfront surgery (hazard ratio (HR): 0.73; *p* *=* *0.025*). There was a clinically relevant improvement of 14 % in the 5-year OS (20.5 % *versus* 6.5 %) for resectable and borderline resectable disease [14]. Although this study has positive results, it has notable limitations due to the use of gemcitabine monotherapy as adjuvant CT, which was the standard of care at the time of the trial design. Building on these early findings, modern studies have adopted more potent CT regimens, such as FOLFIRINOX, combined with advanced RT techniques to refine neoadjuvant strategies.

**Table 2:** Clinical trials evaluating CRT as neoadjuvant setting in the management of BRPC.

Trial	Setting	Treatments	Patient outcomes	Main conclusion
NCT01458717 [36]	Neoadjuvant GEM-based CRT *vs* upfront surgery in patients with BRPC.	Neoadjuvant CRT (45 Gy in 25 fractions and 9 Gy in 5 fractions) (*n* = 27) *vs* upfront surgery with adjuvant CRT (45 Gy in 25 fractions and 9 Gy in 5 fractions) (*n* = 28).	Median OS: CRT 21 months *vs* surgery: 12 months (*p=0.028*). R0 resection rate: CRT 51.8 % *vs* surgery 26.1 %.	Benefits of neoadjuvant GEM-based CRT for BRPC.
PREOPANC (Phase III) [37,14]	Neoadjuvant GEM-based CRT *vs* upfront surgery in RPC and BRPC.	Neoadjuvant GEM-based CRT followed by surgery (3 cycles GEM plus hypofractionated RT of 36 Gy in 15 fractions) (*n* = 119) *vs* upfront surgery (*n* = 127). Both followed by adjuvant GEM (4 or 6 cycles respectively).	Median OS: CRT 15.7 months (95 % CI: 12.9 - 20.6) *vs* upfront surgery: 14.3 months (95 % CI: 12.7 - 17.9) (HR: 0.73; 95 % CI: 0.56 - 0.96; *p=0.025*). 3-year survival: CRT: 27.7 % (95 % CI: 20.7 - 37.1) *vs* upfront surgery: 16.5 % (95 % CI: 11.1 - 24.4). 5-year survival: CRT 20.5 % (95 % CI: 14.2 - 29.8) *vs* upfront surgery 6.5 % (95 % CI: 3.1 - 13.7).	Benefits of GEM-based CRT in long term survival. (Negative initial results for CRT but positive long-term results).
NCT01591733 (Phase II) [39]	Neoadjuvant FFX followed by individualized CAP-based CRT for BRPC.	Neoadjuvant FFX (8 cycles) followed by short-course RT (25 Gy in 5 fractions with proton RT or 30 Gy in 10 fractions with photon RT) (*n* = 27) or long-course RT (50.4 Gy in 28 fractions using IMRT) (*n* = 17).	R0 resection rate: 65 % (95 % CI: 49 - 78). Median PFS: resected patients 48.6 months (95 % CI: 14.4 to NR) *vs* all patients: 14.7 months (95 % CI: 10.5 to NR). 2-year PFS: resected patients 55 % *vs* all patients 43 %. Median OS: resected patients NR *vs* all patients 37.7 months (95 %CI: 19.4 months to NR) 2-year OS: resected patients 72 % *vs* all patients 56 %.	High rates of R0 resection and prolonged median PFS and OS for neoadjuvant FFX followed by individualized CRT.
A021501 (Phase II) [40]	Preoperative mFFX *vs* mFFX plus hypofractionated RT for BRPC.	Neoadjuvant mFFX (*n* = 65) *vs* neoadjuvant mFFX plus RT (SBRT of 33–40 Gy in 5 fractions or hypofractionated image-guided RT of 25 Gy in 5 fractions) (*n* = 55).	Median OS: mFFX 29.8 months (95 % CI, 21.1–36.6) *vs* mFFX plus RT 17.1 months (95 % CI, 12.8 %−24.4). R0 resection: mFFX 28 (88 %) *vs* mFFX plus RT 14 (74 %).	mFFX alone associated to better OS and R0 resection. mFFX plus hypofractionated RT was well tolerated but not effective.
ESPAC-5 (Phase II) [41]	Neoadjuvant GEM plus CAP, FFX or CAP-based CRT in patients with BRPC *vs* immediate surgery.	Immediate surgery (*n* = 33); neoadjuvant GEM plus CAP (*n* = 20); neoadjuvant FFX (*n* = 20); neoadjuvant CAP-based CRT (50.4 Gy in 28 fractions) (*n* = 17).	1-year OS: immediate surgery 39 % (95 % CI: 24 −61); neoadjuvant GEM plus CAP: 78 % (95 % CI: 60 - 100) ; neoadjuvant FFX 84 % (95 % CI: 70 - 100); neoadjuvant CRT 60 % (95 % CI: 37 - 97).	Benefits of neoadjuvant strategy compared to surgery and CT superior to CAP-based CRT. No difference in resection rates.
PREOPANC-2 (Phase III) [42,43]	Total neoadjuvant FFX *vs* neoadjuvant GEM-based CRT and adjuvant GEM for RPC and BRPC.	Neoadjuvant FFX (8 cycles) followed by surgery without adjuvant treatment (*n* = 185) *vs* neoadjuvant GEM (3 cycles) plus hypofractionated RT (36 Gy in 15 fractions) followed by surgery and adjuvant GEM (4 cycles) (*n* = 184).	Median OS: FFX 21.9 months (95 % CI: 17.7 – 27.0) *vs* CRT 21.3 months (95 % CI: 16.8 – 25.5) (HR: 0.87; 95 % CI: 0.68 – 1.12; *p=0.28*).	No difference between FFX and GEM-based CRT in OS. Significant higher N0 for CRT arm.
PANDAS-PRODIGE 44 (Phase II)(44)	Neoadjuvant mFFX with or without preoperative concomitant CRT in BRPC.	Neoadjuvant mFFX followed by surgery and adjuvant CT (GEM or modified LV5FU) (arm A) *vs* neoadjuvant mFFX plus CAP-based CRT (50.4 Gy in 28 fractions) followed by surgery and adjuvant CT (GEM or modified LV5FU) (arm B).	Median OS: arm A 32.8 months (95 % CI: 22.7 – 55.4) *vs* arm B 30 months (95 % CI: 16.5 – nr). R0 resection rate: arm A 54.1 % *vs* arm B 58.1 %.	Conventional CRT did not improve R0 nor OS compared to mFFX without preoperative CRT.

BRPC, borderline resectable pancreatic cancer; CAP, capecitabine; CRT, chemoradiotherapy; CT, chemotherapy; FFX, FOLFIRINOX (combination of 5-FU, leucovorin, irinotecan and oxaliplatin); GEM, gemcitabine; HR, hazard ratio; LV5FU, combination of leucovorin and 5-FU; NR, not reached; OS, overall survival; RPC, resectable pancreatic cancer; RT, radiotherapy.

#### Modern trials and ongoing evidence

Recent evidences have shown that gemcitabine plus capecitabine [10] or FOLFIRINOX [8,38] are superior to gemcitabine monotherapy in adjuvant CT. A single-arm phase II clinical trial was conducted to evaluate the efficacy of neoadjuvant FOLFIRINOX followed by individualized capecitabine-based CRT. CRT was administered based on the restaging of patients after 4 cycles of FOLFIRINOX. If the tumor was clearly resectable, patients received short-course RT with capecitabine (25 Gy in 5 fractions with proton RT or 30 Gy in 10 fractions with photon RT). If the tumor had persistent vascular involvement, patients received long-course RT with capecitabine or 5-FU (50.4 Gy in 28 fractions using intensity modulated radiotherapy (IMRT)) [39]. This study demonstrated that this regimen was associated with high rates of R0 resection, prolonged median PFS and median OS that supporting the pursuit of investigating neoadjuvant FOLFIRINOX followed by CRT. The subsequent phase II study A021501 compared neoadjuvant mFOLFIRINOX with or without hypofractionated RT in 126 patients with BRPC. Patients received either 8 cycles of mFOLFIRINOX or 7 cycles of mFOLFIRINOX followed by SBRT (33–40 Gy in 5 fractions) or hypofractionated image-guided RT (25 Gy in 5 fractions). Patients without disease progression underwent surgery followed by adjuvant CT (FOLFOX6). The primary end point of the 18-month OS rate for both arms was compared to an historical control rate of 50 %. Results showed that neoadjuvant mFOLFIRINOX improved the 18-month OS rate to 66.7 %, while neoadjuvant mFOLFIRINOX followed by RT did not, with a rate of 47.3 %. Pancreatectomy was performed for 49 % of patients with an R0 resection rate of 88 % for neoadjuvant mFOLFIRINOX, while the surgery was performed for 35 % of patients with a 74 % R0 resection rate for neoadjuvant mFOLFIRINOX combined with RT. The authors concluded that neoadjuvant mFOLFIRINOX alone represents a reference regimen for BRPC. However, it was found to be ineffective when combined with hypofractionated RT [40]. Recently the ESPAC-5 phase II trial was conducted to compare three different types of short-course neoadjuvant therapy to immediate surgery. The study enrolled 90 patients with BRPC assigned to immediate surgery (*n* = 33), gemcitabine plus capecitabine (*n* = 20), FOLFIRINOX (*n* = 20) or capecitabine-based CRT (50.4 Gy in 28 fractions) (*n* = 17). There was no significant difference in R0 resection rate between patients who underwent immediate surgery (14 %) and those who received neoadjuvant therapy (23 %; *p* *=* *0.49*). However, the 1-year OS rate was higher for all neoadjuvant therapies: 78 % for gemcitabine plus capecitabine, 84 % for FOLFIRINOX, and 60 % for capecitabine-based CRT, compared to 39 % for immediate surgery (*p* *=* *0.0028*). This study demonstrates that neoadjuvant therapy is favored over immediate surgery in BRPC. Additionally, it indicates that neoadjuvant CT is more effective than CRT [41].

The results of the phase III PREOPANC-2 trial challenged the previously established superiority of neoadjuvant FOLFIRINOX. The study was designed to assess the effectiveness of total neoadjuvant FOLFIRINOX compared with neoadjuvant gemcitabine-based CRT followed by adjuvant gemcitabine in BRPC and resectable patients. Patients were randomly assigned to receive either 8 cycles of FOLFIRINOX followed by surgery without adjuvant therapy (*n* = 185) or 3 cycles of neoadjuvant gemcitabine with hypofractionated RT (36 Gy in 15 fractions), followed by surgery and 4 cycles of adjuvant gemcitabine (*n* = 184). The initial results demonstrated that the median OS was similar between the two groups (21.9 months *versus* 21.3 months; HR 0.87; 95 %CI: 0.68 - 1.12; *p* *=* *0.28*). Additionally, the resection rates were also comparable (77 % *versus* 75 %; *p* *=* *0.69*). However, the gemcitabine-based CRT group exhibited significantly higher node negative (N0) (*p* *<* *0.01*). This trial demonstrated that neoadjuvant CT with FOLFIRINOX did not result in improved survival compared to neoadjuvant gemcitabine-based CRT in patients with borderline resectable and resectable PDAC [42,43].

Currently, the appropriate CT and/or CRT regimen neoadjuvant treatment must be discussed on a case-by-case basis. The most appropriate neoadjuvant strategy for BRPC remains a controversial issue that requires resolution through phase III studies. The results of the phase II French PANDAS-PRODIGE 44 trial (NCT02676349), which aims to evaluate the R0 resection rates with neoadjuvant mFOLFIRINOX followed or not by capecitabine-based CRT (50.4 Gy in 28 fractions) in BRPC, will be of significant interest in resolving the ongoing debate regarding the role of RT in BRPC. The initial findings of this study suggest that the implementation of conventional CRT following neoadjuvant mFOLFIRINOX did not result in a notable enhancement in the R0 resection rate (54.1 % *versus* 58.1 %) nor an improvement in OS (30.0 months *versus* 32.8 months) compared to mFOLFIRINOX monotherapy in patients with BRPC [44]. The final analysis of the clinical trial is currently being pending.

Consequently, for borderline resectable tumors, neoadjuvant treatment is recommended to increase the likelihood of achieving an R0 resection and to improve OS. The role of RT in this setting, however, remains a subject of ongoing debate. If treatment is approved by the tumor board, RT should be delivered after a 2–6-month course of CT [35].

### Locally advanced pancreatic cancer

Patients with LAPC present unresectable disease due to the involvement of vasculature in the tumor (greater than 180° tumor contact with either the superior mesenteric artery (SMA), celiac artery, or involvement of the first jejunal branch of the SMA or aorta). Those patients can benefit from induction therapy to induce the downsizing of the tumor, to reduce the risk of metastases, and hope to obtain a resectable disease. As with other PDAC subtypes, the role of RT in the management of LAPC has been extensively investigated and remains a topic of ongoing debate.

### *Early trials*

A phase III study conducted by the Fédération Francophone de Cancérologie Digestive (FFCD) and the Société Francophone de Radiothérapie Oncologique (SFRO) compared the role of CRT in association with systemic CT to CT alone in 119 patients with LAPC. Patients received intensive induction CRT (60 Gy in 30 fractions with concomitant 5-FU and cisplatin) or induction gemcitabine, and both arms received maintenance gemcitabine. Overall, the survival was shorter in the CRT arm with a median OS of 8.6 months compared to 13 months for the gemcitabine alone (*p* *=* *0.03*) (Table 3). Moreover, patients in the CRT arm experienced significantly more frequent and severe toxic effects [45]. A subsequent study initiated by the Eastern Cooperative Oncology Group explored gemcitabine-based CRT in LAPC. Patients were randomly assigned to receive either gemcitabine alone or in combination with RT (50.4 Gy in 28 fractions). The patients who received CRT had a significantly higher median OS of 11.1 months compared to 9.2 months for those who received gemcitabine alone (*p* *=* *0.017*). The toxicity of gemcitabine-based CRT was acceptable, despite the occurrence of more grade 4 toxicities, primarily comprising fatigue, hematologic, and digestive toxicity [13].

**Table 3:** Clinical trials evaluating CRT as neoadjuvant setting in the management of locally advanced PDAC.

Trial	Setting	Treatments	Patient outcomes	Main conclusion
2000–01 FFCD/SFRO study (Phase III) [45]	Induction CRT followed by maintenance GEM in LAPC.	5-FU plus cisplatin CRT (60 Gy in 30 fractions) induction plus GEM maintenance (*n* = 59) *vs* GEM induction and maintenance (*n* = 60).	Median OS: CRT 8.6 months (99 % CI: 7.1 – 11.4) *vs* GEM 13.0 months (99 % CI:8.7 – 18.1) (*P* = 0.03) (*stratified* log*-rank p = 0.03*).	CRT was more toxic and less effective than GEM alone.
ECOG (Randomized) [13]	GEM alone *vs* GEM plus RT in LAPC.	GEM alone (*n* = 35) *vs* GEM plus RT (50.4 Gy in 28 fractions) (*n* = 34).	Median OS: GEM alone 9.2 months (95 % CI: 7.9 – 11.4) *vs* GEM plus RT 11.1 months (95 % CI: 7.6 – 15.5) (*one-sided p value =0 0.017 by stratified logrank test*).	The addition of RT to GEM improved the OS.
SCALOP (Phase II) [12]	GEM-based or CAP-based consolidation CRT for LAPC.	CAP-based CRT (50.4 Gy in 28 fractions) (*n* = 36) *vs* GEM-based CRT (50.4 Gy in 28 fractions) (*n* = 38) after induction CT (GEM or CAP).	Median OS: CAP-based CRT 15.2 months (95 % CI: 13.9–19.2) *vs* GEM-based CRT 13.4 months (95 % CI: 11.0–15.7) (unadjusted HR: 0.50; 95 % CI: 0.27–0.93; log*-rank p=0·025*/ adjusted HR: 0·39; 95 % CI: 0.18 - 0.81; *p=0.012*).	Better safety and efficacy outcomes for CAP-based CRT than GEM-based CRT.
LAP07 (Phase III) [11]	CRT *vs* CT after induction CT with GEM alone or GEM with Erlo in LAPC.	First randomization: induction CT with GEM alone (*n* = 223) or GEM plus Erlo (*n* = 219). Second randomization: CT (*n* = 136) (GEM alone (*n* = 68) or GEM plus erlotinib (*n* = 68)) *vs* CRT (*n* = 133) (GEM alone and CRT (54 Gy in 30 fractions) (*n* = 67) or GEM plus Erlo and CRT (54 Gy in 30 fractions) (*n* = 66)).	First-randomization: Median OS: GEM alone 13.6 months (95 %CI: 12.3–15.3) *vs* GEM plus erlotinib 11.9 months (95 %CI: 10.4–13.5). Second randomization status: Median OS: CRT 15.2 months (95 %CI: 13.9–17.3) *vs* CT 16.5 months (95 %CI: 14.5–18.5) (HR: 1.03; 95 %CI: 0.79–1.34; *p=0.83*). Locoregional progression: CRT 32 % *vs* CT 46 % (*p=0.03*). Metastatic progression: CRT 60 % than in the CT 44 % (*p = 0.04*).	No survival benefit of CRT compared with CT LAPC controlled after 4 months of induction CT. Efficacy of CRT for local control.
CONKO-007 (Phase III) [47,48]	CRT *vs* CT alone after induction CT (GEM or FFX) in unresectable non metastatic PDAC.	Induction CT with GEM (*n* = 93) or FFX (*n* = 402) followed by CRT (50.4 Gy in 28 fractions) (*n* = 167) or CT (*n* = 168).	Median OS: CT 15 months *vs* CRT 15 months (HR:0.975; 95 % CI:0.756 –1.211; *p=0.713*). Median PFS: CT 8 months *vs* CRT 9 months (HR: 0.976; 95 % CI: 0.780–1.222; *p=0.835*).	CRT increased R0 resection rate and complete pathological response rate in but no significant effect on OS.
NCT03621644 (Phase II) [59,60]	SMART in BRPC or LAPC.	SMART (50 Gy in 5 fractions) (*n* = 136).	2-year OS: 53.6 % from diagnosis and 40.5 % from SMART.	Encouraging OS with SMART, no severe toxicity.
LAP-ABLATE (Phase III) [62]	Induction CT with SMART *vs* CT alone in LAPC.	Induction CT with SMART (50 Gy in 5 fractions) or CT (*n* = 267).	Ongoing.	_
ABLATE (Phase II) [65]	High-dose RT in LAPC with initial response to CT.	High-dose RT (50 Gy in 5 fractions, 67.5 Gy in 15 fractions, or 75 Gy in 25 fractions)	Ongoing	_
LAP100 (Phase III) [66]	Dose-escalated RT *vs* standard treatments in LAPC patients who received prior CT.	Dose-escalated RT (BED of 100 Gy in 5 or 25 fractions) *vs* standard treatments (CT, standard dose CRT, or observation).	Ongoing	_

5-FU, 5-fluorouracil; BED, biological effective dose; BRPC, borderline resectable pancreatic cancer; CAP, capecitabine; CRT, chemoradiotherapy; CT, chemotherapy; DFS, disease-free progression; Erlo, erlotinib; FFX, FOLFIRINOX (combination of 5-FU, leucovorin, irinotecan and oxaliplatin); GEM, gemcitabine; HR, hazard ratio; LAPC, locally advanced pancreatic cancer; LV5FU, combination of leucovorin and 5-FU; OS, overall survival; PFS, progression-free survival; SMART, Stereotactic Magnetic resonance-guided on-table Adaptive Radiation Therapy; RT, radiotherapy.

Other studies have investigated the role of CRT as a consolidation treatment. The phase II SCALOP study was designed to assess the activity, safety, and feasibility of gemcitabine-based and capecitabine-based consolidation CRT (50.4 Gy in 28 fractions) following a course of induction CT in LAPC patients. Although the primary endpoint of PFS was not significantly different, the median OS was significantly improved in capecitabine group compared to gemcitabine (15.2 months *versus* 13.4 months; *p* *=* *0.012*). The authors affirmed that both regimens can be safely administered but capecitabine-based CRT appeared to be the more efficient and the less toxic consolidation treatment. Moreover, grade 3–4 adverse effects were less frequent during consolidation CRT than during induction CT [12]. In a similar vein, the phase III LAP07 trial investigated whether CRT could improve OS in LAPC patients following four months of gemcitabine-based induction CT and to evaluate the effect of erlotinib. The study comprised a first randomization with induction gemcitabine with or without erlotinib. Patients with progression-free disease after four months were then randomized to receive the same CT or capecitabine-based CRT (54 Gy in 30 fractions). The results of both randomizations were negative. Erlotinib and CRT failed to demonstrate any improvement in terms of OS or PFS. Interestingly, the authors reported that locoregional progression was less frequent in the CRT group (32 %) than in the CT group (46 %); *p* *=* *0.03*), whereas metastatic progression was more common in the CRT group (60 %) than in the CT group (44 %; *p* *=* *0.04*) [11]. Although CRT seems to have improved local progression control, it did not improve survival outcomes.

The primary limitation of these earlier studies is that they were designed years before the advent of FOLFIRINOX. A French retrospective study examined the impact of additional preoperative CRT following induction FOLFIRINOX in BRPC and LAPC patients. Both BRPC and LAPC patients who received additional CRT (median dose of 54 Gy in 30 fractions with concurrent 5-FU or capecitabine) exhibited significantly longer OS than those who received FOLFIRINOX alone (57.8 months *versus* 35.5 months; *p* *=* *0.007*). However, the DFS was not significantly different between the two groups. The authors additionally reported other benefits of the addition of CRT, including higher rates of R0 resection and tumor downstaging, and a lower rate of locoregional relapse [46]. The phase III CONKO-007 trial was designed to examine the effectiveness of CRT compared to CT alone after induction CT in LAPC patients. A total of 525 patients received 3 months of induction CT, either gemcitabine (3 cycles) or FOLFIRINOX (6 cycles), depending on the patient’s general health. Subsequently, patients without progression were randomly assigned to receive either the same previous CT regimen for another 3 months or concurrent gemcitabine-based CRT (IMRT with a total dose of 50.4 Gy in 28 fractions). Following a reassessment of the resectability, patients were assigned to either surgery or continued systemic treatment. The authors decided to publish an interim analysis to demonstrate that the resectability of unresectable disease at diagnosis should be re-evaluated after neoadjuvant treatment. Moreover, patients with a high probability of achieving a R0 resection should undergo surgery to improve their prognosis [47]. The initial primary endpoint of the study was OS, but it was subsequently modified to the R0 resection rate due to the delayed accrual of patients. Among the 335 randomized patients, an equal proportion underwent surgery in the CT arm (35.9 %) and in the CRT arm (36.3 %). The R0 circumferential resection margin-negative (CRM-) resection rate was significantly higher in the CRT arm (17 %) than in the CT arm (9.0 %) (*p* *=* *0.0348*). Similarly, the incidence of R1-resections was significantly higher in patients who received CT (10 %) than in those who received CRT (3.0 %) (*p* *=* *0.0133*). The pathological complete response (pCR) was also increased for the patients who received CRT (7 % *versus* 0.6 %; *p* *=* *0.0055*). Despite improving the R0 CRM- and the pCR, the addition of RT after induction CT did not translate into survival benefits. However, the 5-year OS rates displayed a 2.7-fold survival benefit for the CRT arm (9.6 % *versus* 4.3 %) [48].

Early studies have also explored the potential benefits of SBRT for LAPC patients. Institutional studies have demonstrated encouraging outcomes and reported tolerable toxicities, thereby establishing the foundation for the subsequent implementation of randomized clinical trials [[49], [50], [51], [52]].

Collectively, these results appear to indicate that CRT can be effective for the control of the locoregional progression. However, its impact on survival remains limited and requires further investigation. Recognizing the shortcomings of earlier protocols and outdated dosing, recent investigations have explored dose escalation and novel RT delivery methods to improve local control and survival.

### *Modern trials and ongoing evidence*

Conventional fractionated CRT for LAPC has historically been employed to deliver BED ranging from approximately 50–54 Gy (46–60 Gy total doses in 1.8–2.0 Gy per fraction). These conventional-dose RT were established to protect OAR from the adverse effects of prior techniques that utilized large fields. However, advancements in RT techniques which enable a more focused field and sparing of OAR, have rendered biological dose-escalation feasible. Escalated-dose RT, which are typically considered as BED >70 Gy, have been explored in some institutional series, meta-analyses, and prospective phase 1–2 studies, and have demonstrated encouraging results. A single-institution retrospective study has investigated the impact of dose escalation during consolidative CRT following induction CT in LAPC patients. The study revealed that patients who received dose-escalated IMRT with a BED >70 Gy exhibited a significantly improved OS in comparison with those who received BED ≤70 Gy (17.8 *versus* 15.0 months; *p* *= 0.03*). This benefit was sustained during the follow-up period. Furthermore, the dose-escalated group had an improved locoregional recurrence-free survival (10.2 *versus* 6.2 months; *=0.05*) [53]. However, it should be noted that in this study, patients underwent dose escalation exclusively if their tumors were >1 cm away from gastrointestinal mucosal structures. Another retrospective, non-randomized institutional study has investigated the use of ablative RT with a BED of 98 Gy (75 Gy in 25 fractions for tumors <1 cm from the stomach or intestines, and 67.5 Gy in 15 fractions for tumors of 1 cm or more) following multiagent induction therapy in 119 patients with LAPC. The findings of the study indicate that ablative RT was associated with durable locoregional tumor control, as evidenced by a 2-year local tumor progression rate of only 32.8 % and a favorable 2-year OS rate of 38 % from the time of irradiation [54]. The efficacy of ablative RT has been recently demonstrated in patients with localized resectable PDAC who were deemed medically ineligible for surgical resection due to medical comorbidities and advanced age. A total of 25 patients enrolled in the study received a BED of at least 97.5 Gy, and 17 of them received induction CT prior to RT. Ablative RT has been shown to achieve durable control of the primary tumor, with favorable survival outcomes, as evidence by a 2-year OS rate of 43.7 % (95 % CI: 27.4 – 69.5) and a 2-year local progression rate of 20.8 % (95 % CI: 7.3 – 39.0) [55].

Other studies have employed adaptive magnetic resonance imaging (MRI)-guided radiation therapy (MRgRT) to deliver high doses to the tumor while circumventing the OAR. MRgRT integrates a MRI device with a linear accelerator, thereby facilitating real-time visualization of the surrounding organs. This technology enables daily inter-fractional adaptive treatment planning and intra-fractional tumor monitoring. Numerous retrospective studies employing MRgRT have reported promising results and demonstrated the safety of the technique for ablative dose escalation. A preliminary multicenter analysis reported that patients treated with high-dose MRgRT (BED >70 Gy) exhibited a significant improvement in 2-year OS when compared to those receiving standard-dose (BED ≤70 Gy) (49 % *versus* 30 %; *p* *=* *0.03*) [56]. Subsequent single-institution studies have evaluated a MRgRT treatment with 50 Gy in 5 fractions (BED =100 Gy) and demonstrated promising outcomes while minimizing toxicity [57,58].

A paucity of prospective evidence exists to support the escalation of RT dose in LAPC treatment. An initial phase II randomized trial RTOG 1201 was designed to compare conventional-dose RT and escalated-dose RT with concurrent gemcitabine/nab-paclitaxel to gemcitabine/nab-paclitaxel alone in LAPC patients. Unfortunately, the study was prematurely closed due to poor accrual. Nonetheless, a subsequent single-arm prospective phase II trial (NCT03621644) has evaluated Stereotactic Magnetic resonance-guided on-table Adaptive Radiation Therapy (SMART) delivered in 5 fractions with an ablative dose of 50 Gy (BED =100 Gy) in 136 patients with BRPC or LAPC. The preliminary results of the study, with a follow-up of 8.8 months, reported that the primary endpoint was met with grade 3 or higher toxicity occurring in rare cases [59]. However, the authors documented two postoperative deaths that were potentially related to SMART in patients who underwent surgery. Consequently, the authors advise prudence when considering surgery following SMART, particularly in conjunction with vascular resections. Subsequent long-term outcomes of this study confirmed the limited severe toxicity and demonstrated encouraging OS with a 2-year OS rate from diagnosis and from SMART of 53.6 % and 40.5 %, respectively [60]. A recent prospective phase II study has evaluated the use of adaptive MRI-guided SBRT in LAPC patients who received an ablative dose (BED ≥100; 50–60 Gy in 5–8 fractions). The study demonstrated encouraging OS and resection rates, with a median OS from inclusion of 16.5 months (95 % CI: 10.7 – 21.6) and 21 % of patients undergoing resection. The treatment was found to be safe and well tolerated, with limited severe toxicity [61]. While these phase II studies are encouraging, further confirmation is necessary in phase III clinical trials. The ongoing phase III prospective randomized trial, LAP-ABLATE (NCT05585554), aims to confirm the benefits of SMART by comparing induction CT followed by SMART (50 Gy in 5 fractions) with CT alone in LAPC patients [62]. The results of this study are highly anticipated.

Despite its controversial status, the use of escalated-dose RT has exhibited a marked increase as a proportion of pancreas-directed RT, as evidenced in a recent large meta-analysis that reported an escalation from 7 % in 2015 to 22 % in 2019 [63]. The study explored data from 91,493 patients with LAPC, of whom 16,978 patients received definitive pancreas-directed RT and were divided into two groups: those who received conventional-dose RT (BED ≥39 Gy and ≤70 Gy) and those who received escalated-dose RT (BED >70 and ≤132 Gy). The study observed that only a fraction (9 %) of patients received escalated-dose RT, and the median OS estimate for this group was 14.5 months, which was significantly higher than the median estimate of 13.0 months for patients receiving conventional-dose RT (*p* *<* *0.0001*). In addition, multivariable Cox regression analysis revealed that the administration of escalated-dose RT was correlated with a longer OS compared to conventional-dose RT (adjusted hazard ratio: 0.85; 95 % CI: 0.80–0.91; *p* *<* *0.001*).

Prospective studies are underway to investigate the efficacy of high-dose RT in LAPC patients. For instance, the phase II trial ABLATE (NCT06453486) will assess the efficacy of high-dose RT (50 Gy in 5 fractions, 67.5 Gy in 15 fractions, or 75 Gy in 25 fractions) in patients with LAPC who have demonstrated a response to initial CT. Another recent phase III randomized controlled trial, LAP100 (NCT06958328), was designed to assess the role of dose-escalated RT to usual care in LAPC patients. This trial will enroll patients who received an initial 4–6 months of CT with FOLFIRINOX, NALIRIFOX (fluorouracil, liposomal irinotecan, leucovorin, and oxaliplatin), or gemcitabine/nab-paclitaxel. Following enrollment, patients will be randomly assigned to receive either dose-escalated RT (BED of 100 Gy in 5 or 25 fractions) or standard of care treatment (additional CT, standard dose CRT or observation) at the discretion to the physician. With a target accrual of 356 patients, the study will strongly determine if dose-escalated RT improves the 3-year OS of LAPC patients.

The advent of conformal treatment methods, image guidance, and motion management techniques holds great promise in ensuring the safe intensification of RT treatment, thereby improving radiation outcomes for LAPC patients.

Induction CT followed by CRT may be considered for selected patients who show no disease progression after at least 3 months of systemic therapy, with a potential benefit of dose escalation currently under investigation [64].

## Future directions

Significant research has been conducted over the past few decades to investigate the potential of RT in the treatment of PDAC. A range of clinical trials have assessed the integration of RT in different clinical contexts. However, their interpretation is often limited by insufficient technical details, the use of outdated CT and RT regimens- such as split-course and low-dose approaches – and heterogeneous patient selection. In particular, several studies have included a mixture of patients diagnosed with both BRPC and LAPC, although these diseases have distinct characteristics and prognoses. This heterogeneity complicates comparisons between studies and hinders the establishment of clear clinical recommendations. The absence of consensus regarding the optimal RT modalities represents a crucial area for investigation in future clinical trials.

Before addressing upcoming innovations and future clinical application, it is essential to recall the current standards for RT planning and delivery, which provide the foundation for treatment accuracy and reproducibility. Current RT procedures for acquisition protocol and motion management recommend that patients should be positioned supine with their arms raised above the head. The utilization of immobilization devices, such as leg rests, footrests, thermoplastic shells, or vacuum mattresses is strongly recommended to ensure the reproducibility. Prior to simulation, patients should fast for 3–4 h, and treatment should be delivered under identical conditions. Subsequent to a non-contrast scan, contrast-enhanced acquisitions are obtained under the same breathing conditions (free breathing for standard delivery or respiratory-gated for SBRT). Two to three contrast phases are recommended to ensure optimal visualization of the tumor and adjacent structures. A four-dimensional computed tomography (4D CT) is strongly recommended to assess respiratory motion, though diaphragmatic compression may be used as an alternative. In regard to target volume delineation, dose prescription, and dosimetric planning, it is imperative to refer to the various international recommendations published to date [64,67,68]. Daily image guidance is recommended for both IMRT and SBRT. Building on these standardized approaches, current evidence indicates that RT may provide selective benefits depending on disease stage and patient profile.

The extant evidence supports that adjuvant RT or CRT may offer an advantage only in specific patient subsets, such as those with node-positive disease [69] or those at high risk of local recurrence, as recommended in the latest NCCN Guidelines. Nevertheless, the potential of RT in the neoadjuvant setting remains under investigation but appears promising in terms of locoregional control. Recent advances in RT techniques, including SBRT, MRgRT, and SMART, have led to a shift in the RT paradigm for PDAC. These techniques enable the delivery of escalated-dose RT to the tumor while sparing the surrounding organs, making them particularly attractive for LAPC management. Unlike conventional RT, SBRT can be completed within a shorter duration and has the capacity to deliver a higher dose per fraction to a smaller target volume. This results in better tolerability for patients and a decrease in time away from full dose of CT. However, the current utilization of SBRT remains restricted to BRPC and LAPC cases, excluding large tumors [70]. According to NCCN Guidelines, no specific SBRT dosing recommendation currently exists; SBRT should ideally be performed in an experienced high-volume center or within clinical trials. A number of ongoing clinical trials are underway to determine the most effective parameters for implementing SBRT in the management of patients with PDAC.

Other innovative approaches for improving the efficacy of RT involve the use of radiosensitizing agents designed to increase RT-induced DNA damage, inhibit repair mechanisms, or impair the components associated with RT resistance. These radiosensitizers include molecules that target DNA damage response or cell cycle checkpoints, as well as high-atomic number nanoparticles [71]. Another potential strategy entails enhancing the immunomodulatory effect of RT by employing immunotherapy. Following promising outcomes in preclinical and early clinical trials results, the combination of RT with immune checkpoint inhibitors (targeting PD-1, PD-L1 and CTLA-4) is under active investigation in multiple ongoing PDAC trials [[72], [73], [74], [75]].

Enhancing RT effects may also depend on identifying predictive biomarkers to better select patients most likely to benefit. A number of studies have reported the potential of serum biomarkers, such as carbohydrate antigen 19–9 (*CA* 19–9), which have elevated levels associated with unfavorable patient outcomes following the completion of CRT [[76], [77], [78], [79], [80], [81]]. Subsequent studies will employ innovative strategies to predict RT response. The use of patient-derived organoids to model the response of PDAC to RT, in conjunction with online omics databases with tailored bioinformatic tools represents a rapid and promising avenue for identifying candidate biomarkers [[82], [83], [84], [85]].

Collectively, these advances highlight a paradigm shift in RT for PDAC, setting the stage for future trials to define its optimal role.

## Conclusion

Despite decades of research, the role of RT in the management of PDAC remains controversial. Historical clinical trials have yielded conflicting results, partly due to the use of outdated RT techniques, heterogeneous patient selection, and varied treatment regimens. Recent evidence suggests that RT, whether in the adjuvant or neoadjuvant setting, may be beneficial for specific patient subgroups, particularly those at high risk of local recurrence or nodal involvement.

Technical advances such as SBRT, MRgRT and SMART now allow safe dose escalation, improving locoregional control while concurrently limiting toxicity. Moreover, combining RT with radiosensitizers, immunotherapy, and predictive biomarkers offers further opportunities to tailor treatment.

To definitely establish the value of RT in PDAC, well-designed prospective trials employing modern RT protocols and carefully stratified cohorts are essential. The optimal integration of RT with contemporary CT regimens and multimodal approaches has the potential to transform it from a debated option into a cornerstone of PDAC management for appropriately selected patients.

## CRediT authorship contribution statement

**Julie Dardare:** Writing – review & editing, Writing – original draft, Conceptualization. **Nicolas Martz:** Writing – review & editing, Resources. **Andréa Witz:** Writing – review & editing. **Margaux Betz:** Writing – review & editing. **Cassandra Michel:** Writing – review & editing. **Pauline Gilson:** Supervision. **Jean-Louis Merlin:** Supervision. **Aurélien Lambert:** Writing – review & editing. **Alexandre Harle:** Writing – review & editing, Supervision, Conceptualization.

## Declaration of competing interest

The authors declare that they have no known competing financial interests or personal relationships that could have appeared to influence the work reported in this paper.

## References

[1] Siegel R.L., Giaquinto A.N., Jemal A. (2024). Cancer statistics, 2024. CA Cancer J. Clin..

[2] Al-Hawary M.M., Francis I.R., Chari S.T., Fishman E.K., Hough D.M., Lu D.S. (2014 Jan). Pancreatic ductal adenocarcinoma radiology reporting template: consensus statement of the society of abdominal radiology and the american pancreatic association. Gastroenterology.

[3] Lambert A., Schwarz L., Borbath I., Henry A., Van Laethem J.L., Malka D. (2019). An update on treatment options for pancreatic adenocarcinoma. Ther. Adv. Med. Oncol..

[4] Burris H.A., Moore M.J., Andersen J., Green M.R., Rothenberg M.L., Modiano M.R. (1997 Jun). Improvements in survival and clinical benefit with gemcitabine as first-line therapy for patients with advanced pancreas cancer: a randomized trial. J. Clin. Oncol. Off. J. Am. Soc. Clin. Oncol..

[5] Neoptolemos J.P., Stocken D.D., Friess H., Bassi C., Dunn J.A., Hickey H. (2004 Mar 18). A randomized trial of chemoradiotherapy and chemotherapy after resection of pancreatic cancer. N. Engl. J. Med..

[6] Conroy Desseigne F, Ychou M., Bouché O., Guimbaud R., Bécouarn Y. (2011 May 12). FOLFIRINOX *versus* Gemcitabine for metastatic pancreatic cancer. N. Engl. J. Med..

[7] Conroy Gavoille C, Samalin E., Ychou M., Ducreux M. (2013 Apr). The role of the FOLFIRINOX regimen for advanced pancreatic cancer. Curr. Oncol. Rep..

[8] Conroy Hammel, Pascal Hebbar, Mohamed Ben Abdelghani, Meher Wei, Alice C., Jean-Luc Raoul (2018 Dec 20). FOLFIRINOX or Gemcitabine as adjuvant therapy for pancreatic cancer. N. Engl. J. Med..

[9] Von Hoff D.D., Ervin T., Arena F.P., Chiorean E.G., Infante J., Moore M. (2013 Oct 31). Increased survival in pancreatic cancer with nab-Paclitaxel plus Gemcitabine. N. Engl. J. Med..

[10] Neoptolemos J.P., Palmer D.H., Ghaneh P., Psarelli E.E., Valle J.W., Halloran C.M. (2017 Mar 11). Comparison of adjuvant gemcitabine and capecitabine with gemcitabine monotherapy in patients with resected pancreatic cancer (ESPAC-4): a multicentre, open-label, randomised, phase 3 trial. Lancet.

[11] Hammel P., Huguet F., van Laethem J.L., Goldstein D., Glimelius B., Artru P. (2016 May 3). Effect of chemoradiotherapy *vs* chemotherapy on survival in patients with locally advanced pancreatic cancer controlled after 4 months of Gemcitabine with or without Erlotinib: the LAP07 randomized clinical trial. JAMa.

[12] Mukherjee S., Hurt C.N., Bridgewater J., Falk S., Cummins S., Wasan H. (2013 Apr). Gemcitabine-based or capecitabine-based chemoradiotherapy for locally advanced pancreatic cancer (SCALOP): a multicentre, randomised, phase 2 trial. Lancet Oncol..

[13] Loehrer P.J., Feng Y., Cardenes H., Wagner L., Brell J.M., Cella D. (2011 Nov 1). Gemcitabine alone *versus* gemcitabine plus radiotherapy in patients with locally advanced pancreatic cancer: an Eastern Cooperative Oncology Group trial. J. Clin. Oncol. Off. J. Am. Soc. Clin. Oncol..

[14] Versteijne E., van Dam J.L., Suker M., Janssen Q.P., Groothuis K., Akkermans-Vogelaar J.M. (2022 Apr 10). Neoadjuvant chemoradiotherapy *versus* upfront surgery for resectable and borderline resectable pancreatic cancer: long-term results of the Dutch randomized PREOPANC trial. J. Clin. Oncol. Off. J. Am. Soc. Clin. Oncol..

[15] Conroy Pfeiffer P, Vilgrain V., Lamarca A., Seufferlein T., O’Reilly E.M. (2023 Nov). Pancreatic cancer: ESMO Clinical Practice Guideline for diagnosis, treatment and follow-up. Ann. Oncol. Off. J. Eur. Soc. Med. Oncol..

[16] Khorana A.A., McKernin S.E., Berlin J., Hong T.S., Maitra A., Moravek C. (2019 Aug 10). Potentially curable pancreatic adenocarcinoma: ASCO clinical practice Guideline update. J. Clin. Oncol..

[17] Balaban E.P., Mangu P.B., Khorana A.A., Shah M.A., Mukherjee S., Crane C.H. (2016 Aug 1). Locally advanced, unresectable pancreatic cancer: american Society of Clinical Oncology Clinical Practice Guideline. J. Clin. Oncol..

[18] Kaiser M.H., Ellenberg S.S. (1985 Aug 1). Pancreatic cancer: adjuvant combined radiation and chemotherapy following curative resection. Arch. Surg..

[19] Group Gastrointestinal Tumor Study (1987 Jun 15). Further evidence of effective adjuvant combined radiation and chemotherapy following curative resection of pancreatic cancer. gastrointestinal tumor study group. Cancer.

[20] Klinkenbijl J.H., Jeekel J., Sahmoud T., van Pel R., Couvreur M.L., Veenhof C.H. (1999 Dec). Adjuvant radiotherapy and 5-fluorouracil after curative resection of cancer of the pancreas and periampullary region: phase III trial of the EORTC gastrointestinal tract cancer cooperative group. Ann. Surg..

[21] Regine W.F., Winter K.A., Abrams R.A., Safran H., Hoffman J.P., Konski A. (2008 Mar 5). Fluorouracil *vs* gemcitabine chemotherapy before and after fluorouracil-based chemoradiation following resection of pancreatic adenocarcinoma: a randomized controlled trial. JAMa.

[22] Regine W.F., Winter K.A., Abrams R., Safran H., Hoffman J.P., Konski A. (2011 May 1). Fluorouracil-based chemoradiation with either Gemcitabine or Fluorouracil chemotherapy after resection of pancreatic adenocarcinoma: 5-year analysis of the U.S. Intergroup/RTOG 9704 Phase III trial. Ann. Surg. Oncol..

[23] Schmidt J., Abel U., Debus J., Harig S., Hoffmann K., Herrmann T. (2012 Nov 20). Open-label, multicenter, randomized Phase III trial of adjuvant chemoradiation plus interferon alfa-2b *versus* fluorouracil and folinic acid for patients with resected pancreatic adenocarcinoma. J. Clin. Oncol..

[24] Hsu C.C., Herman J.M., Corsini M.M., Winter J.M., Callister M.D., Haddock M.G. (2010 Apr 1). Adjuvant chemoradiation for pancreatic adenocarcinoma: the Johns Hopkins hospital—mayo clinic collaborative study. Ann. Surg. Oncol..

[25] Corsini M.M., Miller R.C., Haddock M.G., Donohue J.H., Farnell M.B., Nagorney D.M. (2008 Jul 20). Adjuvant radiotherapy and chemotherapy for pancreatic carcinoma: the Mayo Clinic experience (1975-2005). J. Clin. Oncol..

[26] Wulaningsih W., Ah-Moye A., Khan A., Senthivel N., Tait D.M., Chong I.Y.S. (2025 Feb). Clinical outcomes of adjuvant chemoradiotherapy in pancreatic adenocarcinoma with R1 resection. J. Clin. Oncol..

[27] Garofalo M.C., Regine W.F., Tan M.T (2006). On statistical reanalysis, the EORTC trial is a positive trial for adjuvant chemoradiation in pancreatic cancer. Ann. Surg..

[28] Abrams R.A., Winter K.A., Safran H., Goodman K.A., Regine W.F., Berger A.C. (2020). Results of the NRG oncology/RTOG 0848 adjuvant chemotherapy question-Erlotinib+Gemcitabine for resected cancer of the pancreatic head: a phase II randomized clinical trial. Am. J. Clin. Oncol..

[29] Abrams R.A., Winter K.A., Goodman K.A., Regine W., Safran H., Berger A.C. (2024). NRG Oncology/RTOG 0848: results after adjuvant chemotherapy ± chemoradiation for patients with resected periampullary pancreatic adenocarcinoma (PA). J. Clin. Oncol..

[30] Rwigema J.C.M., Heron D.E., Parikh S.D., Zeh H.J., Moser J.A., Bahary N. (2012). Adjuvant stereotactic body radiotherapy for resected pancreatic adenocarcinoma with close or positive margins. J. Gastrointest. Cancer.

[31] Bernard M.E., Sutera P.A., Iarrobino N.A., Quan K., Burton S.A., Bahary N. (2019). Initial results of a prospective study of adjuvant pancreatic stereotactic body radiation therapy for close or positive margins. Adv. Radiat. Oncol..

[32] Ma T., Bai X., Wei Q., Shui Y., Lao M., Chen W. (2022). Adjuvant therapy with gemcitabine and stereotactic body radiation therapy *versus* gemcitabine alone for resected stage II pancreatic cancer: a prospective, randomized, open-label, single center trial. BMC Cancer.

[33] Istituto Clinico Humanitas. Stereotactic pancreatic radiotherapy adjuvant therapy (SPARTA): phase II Trial. [Internet]. clinicaltrials.Gov; 2025 Apr [cited 2025 Aug 21]. Report No.: NCT05043857. Available from: https://clinicaltrials.gov/study/NCT05043857.

[34] Stocken D.D., Büchler M.W., Dervenis C., Bassi C., Jeekel H., Klinkenbijl J.H.G. (2005). Meta-analysis of randomised adjuvant therapy trials for pancreatic cancer. Br. J. Cancer.

[35] Palta M., Godfrey D., Goodman K.A., Hoffe S., Dawson L.A., Dessert D. (2019). Radiation Therapy for pancreatic cancer: executive summary of an ASTRO clinical practice guideline. Pract. Radiat. Oncol..

[36] Jang J.Y., Han Y., Lee H., Kim S.W., Kwon W., Lee K.H. (2018). Oncological benefits of neoadjuvant chemoradiation with Gemcitabine *versus* upfront surgery in patients with borderline resectable pancreatic cancer: a prospective, randomized, open-label, Multicenter Phase 2/3 trial. Ann. Surg..

[37] Versteijne E., Suker M., Groothuis K., Akkermans-Vogelaar J.M., Besselink M.G., Bonsing B.A. (2020). Preoperative chemoradiotherapy *versus* immediate surgery for resectable and borderline resectable pancreatic cancer: results of the Dutch randomized phase III PREOPANC trial. J. Clin. Oncol. Off. J. Am. Soc. Clin. Oncol..

[38] Conroy Castan F, Lopez A., Turpin A., Ben Abdelghani M., Wei A.C. (2022). Five-year outcomes of FOLFIRINOX *vs* Gemcitabine as adjuvant therapy for pancreatic cancer: a randomized clinical trial. JAMa Oncol..

[39] Murphy J.E., Wo J.Y., Ryan D.P., Jiang W., Yeap B.Y., Drapek L.C. (2018). Total neoadjuvant therapy with FOLFIRINOX followed by individualized chemoradiotherapy for borderline resectable pancreatic adenocarcinoma: a phase 2 clinical trial. JAMa Oncol..

[40] Katz M.H.G., Shi Q., Meyers J., Herman J.M., Chuong M., Wolpin B.M. (2022). Efficacy of preoperative mFOLFIRINOX *vs* mFOLFIRINOX Plus hypofractionated radiotherapy for borderline resectable adenocarcinoma of the pancreas: the A021501 phase 2 randomized clinical trial. JAMa Oncol..

[41] Ghaneh P., Palmer D., Cicconi S., Jackson R., Halloran C.M., Rawcliffe C. (2023). Immediate surgery compared with short-course neoadjuvant gemcitabine plus capecitabine, FOLFIRINOX, or chemoradiotherapy in patients with borderline resectable pancreatic cancer (ESPAC5): a four-arm, multicentre, randomised, phase 2 trial. Lancet Gastroenterol. Hepatol..

[42] Janssen Q.P., van Dam J.L., Bonsing B.A., Bos H., Bosscha K.P., Coene P.P.L.O. (2021). Total neoadjuvant FOLFIRINOX *versus* neoadjuvant gemcitabine-based chemoradiotherapy and adjuvant gemcitabine for resectable and borderline resectable pancreatic cancer (PREOPANC-2 trial): study protocol for a nationwide multicenter randomized controlled trial. BMC. Cancer.

[43] Groot Koerkamp B., Janssen Q.P., van Dam J.L., Bonsing B.A., Bos H., Bosscha K.P. (2023). Neoadjuvant chemotherapy with FOLFIRINOX *versus* neoadjuvant gemcitabine-based chemoradiotherapy for borderline resectable and resectable pancreatic cancer (PREOPANC-2): a multicenter randomized controlled trial. Ann. Oncol..

[44] Lambert A., Bouche O., Ayav A., Bachet J.B., Schwarz L., Piessen G. (2024). Preoperative modified FOLFIRINOX (mFOLFIRINOX) with or without chemoradiation (CRT) in borderline resectable pancreatic cancer (BRPC): results fro. Ann. Oncol..

[45] Chauffert B., Mornex F., Bonnetain F., Rougier P., Mariette C., Bouché O. (2008). Phase III trial comparing intensive induction chemoradiotherapy (60 Gy, infusional 5-FU and intermittent cisplatin) followed by maintenance gemcitabine with gemcitabine alone for locally advanced unresectable pancreatic cancer. Definitive results of the 2000–01 FFCD/SFRO study. Ann. Oncol..

[46] Pietrasz D., Turrini O., Vendrely V., Simon J.M., Hentic O., Coriat R. (2019). How does chemoradiotherapy following induction FOLFIRINOX improve the results in resected borderline or locally advanced pancreatic adenocarcinoma? An AGEO-FRENCH multicentric cohort. Ann. Surg. Oncol..

[47] Fietkau R., Grützmann R., Wittel U.A., Croner R.S., Jacobasch L., Neumann U.P. (2021). R0 resection following chemo (radio)therapy improves survival of primary inoperable pancreatic cancer patients. Interim results of the German randomized CONKO-007± trial. Strahlenther Onkol. Organ. Dtsch. Rontgengesellschaft Al.

[48] Fietkau R., Ghadimi M., Grützmann R., Wittel U.A., Jacobasch L., Uhl W. (2022). Randomized phase III trial of induction chemotherapy followed by chemoradiotherapy or chemotherapy alone for nonresectable locally advanced pancreatic cancer: first results of the CONKO-007 trial. J. Clin. Oncol..

[49] Mahadevan A., Jain S., Goldstein M., Miksad R., Pleskow D., Sawhney M. (2010). Stereotactic body radiotherapy and gemcitabine for locally advanced pancreatic cancer. Int. J. Radiat. Oncol. Biol. Phys..

[50] Zhong J., Patel K., Switchenko J., Cassidy R.J., Hall W.A., Gillespie T. (2017). Outcomes for patients with locally advanced pancreatic adenocarcinoma treated with stereotactic body radiation therapy *versus* conventionally fractionated radiation. Cancer.

[51] Schellenberg D., Kim J., Christman-Skieller C., Chun C.L., Columbo L.A., Ford J.M. (2011). Single-fraction stereotactic body radiation therapy and sequential gemcitabine for the treatment of locally advanced pancreatic cancer. Int. J. Radiat. Oncol..

[52] Moningi S., Dholakia A.S., Raman S.P., Blackford A., Cameron J.L., Le D.T. (2015). The role of stereotactic body radiation therapy for pancreatic cancer: a single-institution experience. Ann. Surg. Oncol..

[53] Krishnan S., Chadha A.S., Suh Y., Chen H.C., Rao A., Das P. (2016). Focal radiation therapy dose escalation improves overall survival in locally advanced pancreatic cancer patients receiving induction chemotherapy and consolidative chemoradiation. Int. J. Radiat. Oncol. Biol. Phys..

[54] Reyngold M., O’Reilly E.M., Varghese A.M., Fiasconaro M., Zinovoy M., Romesser P.B. (2021). Association of ablative radiation therapy with survival among patients with inoperable pancreatic cancer. JAMa Oncol..

[55] Reyngold M., Schoenfeld J.D., O’Reilly E.M., Varghese A.M., White C., Zinovoy M. (2025). Nonoperative management of technically resectable pancreatic cancer with ablative radiation therapy. JAMa Oncol..

[56] Rudra S., Jiang N., Rosenberg S.A., Olsen J.R., Roach M.C., Wan L. (2019). Using adaptive magnetic resonance image-guided radiation therapy for treatment of inoperable pancreatic cancer. Cancer Med..

[57] Hassanzadeh C., Rudra S., Bommireddy A., Hawkins W.G., Wang-Gillam A., Fields R.C. (2021). Ablative five-fraction stereotactic body radiation therapy for inoperable pancreatic cancer using online MR-guided adaptation. Adv. Radiat. Oncol..

[58] Chuong M.D., Herrera R., Chundru S., Gutierrez A., Romaguera T., Alvarez D. (2021). Cumulative target volume dose and locoregional failure in pancreatic cancer patients with treated with ablative stereotactic MR-guided adaptive radiation therapy (SMART). Int. J. Radiat. Oncol..

[59] Parikh P.J., Lee P., Low D.A., Kim J., Mittauer K.E., Bassetti M.F. (2023). A multi-institutional phase 2 trial of ablative 5-fraction stereotactic magnetic resonance-guided on-table adaptive radiation therapy for borderline resectable and locally advanced pancreatic cancer. Int. J. Radiat. Oncol. Biol. Phys..

[60] Chuong M.D., Lee P., Low D.A., Kim J., Mittauer K.E., Bassetti M.F. (2024). Stereotactic MR-guided on-table adaptive radiation therapy (SMART) for borderline resectable and locally advanced pancreatic cancer: a multi-center, open-label phase 2 study. RadiOther Oncol..

[61] Weisz Ejlsmark M., Bahij R., Schytte T., Rønn Hansen C., Bertelsen A., Mahmood F. (2024). Adaptive MRI-guided stereotactic body radiation therapy for locally advanced pancreatic cancer - A phase II study. Radiother. Oncol. J. Eur. Soc. Ther. Radiol. Oncol..

[62] Viewray Inc (2022). https://clinicaltrials.gov/study/NCT05585554.

[63] Shi C., De B., Tran Cao H.S., Liu S., Florez M.A., Kouzy R. (2024). Escalated-dose radiotherapy for unresected locally advanced pancreatic cancer: patterns of care and survival in the United States. Cancer Med..

[64] Bertrand-Chevrier M., Riou O., Orthuon A., Bessières I., Jaksic N., Guimas V. (2025). Radiotherapy of pancreatic cancers: 2025 update. Cancer Radiother. J. Soc. Francaise Radiother. Oncol..

[65] (2025). Study details | NCT06453486 | A Study of high dose radiation therapy for locally advanced pancreatic cancer that responded to initial chemotherapy treatment | ClinicalTrials.Gov [Internet]. NCT06453486.

[66] (2025). Study details | NCT06958328 | testing higher dose radiation therapy for locally advanced pancreatic cancer | ClinicalTrials.Gov [Internet]. NCT06958328.

[67] Goodman K.A., Regine W.F., Dawson L.A., Ben-Josef E., Haustermans K., Bosch W.R. (2012). Radiation therapy oncology group consensus panel guidelines for the delineation of the clinical target volume in the postoperative treatment of pancreatic head cancer. Int. J. Radiat. Oncol. Biol. Phys..

[68] Sanford N.N., Narang A.K., Aguilera T.A., Bassetti M.F., Chuong M.D., Erickson B.A. (2025). NRG Oncology international consensus contouring atlas on target volumes and dosing strategies for dose-escalated pancreatic cancer radiation therapy. Int. J. Radiat. Oncol. Biol. Phys..

[69] Xiao X., Huang P., Xu X.T. (2025). The choice of adjuvant radiotherapy in pancreatic cancer patients after up-front radical surgery. PLoS. One.

[70] Burkoň P., Trna J., Slávik M., Němeček R., Kazda T., Pospíšil P. (2022). Stereotactic body radiotherapy (SBRT) of pancreatic cancer—A critical review and practical consideration. Biomedicines.

[71] Dardare J., Martz N., Witz A., Betz M., Michel C., Gilson P. (2025). Improving radiation therapy in pancreatic cancer through radiosensitization strategies. Int. J. Radiat. Oncol. Biol. Phys..

[72] Azad A., Yin Lim S., D’Costa Z., Jones K., Diana A., Sansom O.J. (2017). PD-L1 blockade enhances response of pancreatic ductal adenocarcinoma to radiotherapy. EMBo Mol. Med..

[73] Xie C., Duffy A.G., Brar G., Fioravanti S., Mabry-Hrones D., Walker M. (2020). Immune checkpoint blockade in combination with stereotactic body radiotherapy in patients with metastatic pancreatic ductal adenocarcinoma. Clin. Cancer Res. Off. J. Am. Assoc. Cancer Res..

[74] Chen I.M., Johansen J.S., Theile S., Hjaltelin J.X., Novitski S.I., Brunak S. (2022). Randomized phase II study of Nivolumab with or without Ipilimumab combined with stereotactic body radiotherapy for refractory metastatic pancreatic cancer (CheckPAC). J. Clin. Oncol. Off. J. Am. Soc. Clin. Oncol..

[75] Du J., Lu C., Mao L., Zhu Y., Kong W., Shen S. (2023). PD-1 blockade plus chemoradiotherapy as preoperative therapy for patients with BRPC/LAPC: a biomolecular exploratory, phase II trial. Cell Rep. Med..

[76] Takahashi H., Ohigashi H., Ishikawa O., Eguchi H., Gotoh K., Yamada T. (2010). Serum CA19-9 alterations during preoperative gemcitabine-based chemoradiation therapy for resectable invasive ductal carcinoma of the pancreas as an indicator for therapeutic selection and survival. Ann. Surg..

[77] Koom W.S., Seong J., Kim Y.B., Pyun H.O., SY Song (2009). *CA* 19-9 as a predictor for response and survival in advanced pancreatic cancer patients treated with chemoradiotherapy. Int. J. Radiat. Oncol. Biol. Phys..

[78] Micke O., Bruns F., Kurowski R., Horst E., deVries A.F., Hausler J.W. (2003). Predictive value of carbohydrate antigen 19-9 in pancreatic cancer treated with radiochemotherapy. Int. J. Radiat. Oncol. Biol. Phys..

[79] Vainshtein J.M., Schipper M., Zalupski M.M., Lawrence T.S., Abrams R., Francis I.R. (2013). Prognostic significance of carbohydrate antigen 19-9 in unresectable locally advanced pancreatic cancer treated with dose-escalated intensity modulated radiation therapy and concurrent full-dose gemcitabine: analysis of a prospective phase 1/2 dose escalation study. Int. J. Radiat. Oncol. Biol. Phys..

[80] Yoo T., Lee W.J., Woo S.M., Kim T.H., Han S.S., Park S.J. (2011). Pretreatment carbohydrate antigen 19-9 level indicates tumor response, early distant metastasis, overall survival, and therapeutic selection in localized and unresectable pancreatic cancer. Int. J. Radiat. Oncol. Biol. Phys..

[81] Golden D.W., Novak C.J., Minsky B.D., Liauw S.L. (2012). Radiation dose ≥54 gy and *CA* 19-9 response are associated with improved survival for unresectable, non-metastatic pancreatic cancer treated with chemoradiation. Radiat. Oncol. Lond Engl..

[82] Shukla H.D., Dukic T., Roy S., Bhandary B., Gerry A., Poirier Y. (2022). Pancreatic cancer derived 3D organoids as a clinical tool to evaluate the treatment response. Front. Oncol..

[83] Naumann M., Czempiel T., Lößner A.J., Pape K., Beyreuther E., Löck S. (2022). Combined systemic drug treatment with proton therapy: investigations on patient-derived organoids. Cancers. (Basel).

[84] Nicosia L., Alongi F., Andreani S., Ruggieri R., Rusev B., Mantoan B. (2020). Combinatorial effect of magnetic field and radiotherapy in PDAC organoids: a pilot study. Biomedicines..

[85] Caxali G.H., Brugnerotto L., Aal M.C.E., Castro C.F.B. (2023). Delella FK. Identification of biomarkers related to the efficacy of radiotherapy in pancreatic cancer. Cancer Genomics. Proteomics..

